# Role of miRNAs on the Pathophysiology of Cardiovascular Diseases

**DOI:** 10.5935/abc.20180215

**Published:** 2018-11

**Authors:** Debora Cristina Pereira da Silva, Felipe Demani Carneiro, Kelly Costa de Almeida, Caroline Fernandes-Santos

**Affiliations:** 1 Programa de Pós-graduação em Ciências Cardiovasculares da Universidade Federal Fluminense (UFF), Niterói, RJ - Brazil; 2 Universidade Federal Fluminense (UFF), Nova Friburgo, RJ - Brazil

**Keywords:** Cardiovascular Diseases/physiopathology, Cardiovascular Diseases/diagnosis, Cardiovascular Diseases/genetics, Biomarkers/metabolism, Cardiac, Remodeling/genetics, Atherosclerosis, MicroRNAs

## Abstract

MiRNA (or microRNA) is a subclass of non-coding RNAs that is responsible for post-transcriptional gene regulation. It has approximately 22 nucleotides and regulates gene expression in plants and animals at the post-transcriptional level, by the cleavage of a target mRNA or by suppression of its translation. Although many of the processes and mechanisms have not yet been fully elucidated, there is a strong association between miRNA expression and several diseases. It is known that miRNAs are expressed in the cardiovascular system, but their role in cardiovascular diseases (CVDs) has not been clearly established. In this non-systematic review of the literature, we first present the definition of miRNAs and their action at the cellular level. Afterward, we discuss the role of miRNAs as circulating biomarkers of CVDs, and then their role in cardiac remodeling and atherosclerosis. Despite the complexity and challenges, it is crucial to identify deregulated miRNAs in CVDs, as it allows a better understanding of underlying cellular and molecular mechanisms and helps in the development of more accurate diagnostic and prognostic circulating biomarkers, and new therapeutic strategies for different stages of CVDs.

## Introduction

Scientific research has been done in attempts to explain the pathophysiology of several diseases for the development of new therapies. In this regard, miRNA (or microRNA) has drawn attention of scientific community as a potential therapeutic target. Since their discovery in 1993,^1^ several miRNAs related to biologic processes for their gene-regulatory roles have been cataloged. However, many miRNAs remain to be discovered, which makes them one of the largest classes of gene regulators. To give an idea of the importance of miRNAs, these molecules regulate approximately one third of all gene expression in mammals.^2^

Although many studies have successfully established an association between miRNA expression patterns and several diseases, many mechanisms and processes involved have not been fully elucidated. In diabetes mellitus, for example, results of experimental studies have indicated that specific miRNAs present in pancreatic islets may play a regulatory role in insulin secretion.^3^ MiRNA expression may also be visualized in different types of tumors, acting either as tumor suppressors or exerting an opposite role with deleterious effects.^4^ Although it is currently known that miRNAs are expressed in the cardiovascular system, their role on the development of cardiovascular diseases (CVDs) still need to be better understood.

In light of this, we conducted a systematic review aimed at summarizing and discussing the findings of the main studies investigating the relationship between miRNAs and CVD. We searched for articles published in the PubMed database (www.ncbi.nlm.nih.gov/pubmed). Original articles written in English, involving humans or animals, were selected using the following MeSH terms - microRNA AND Cardiovascular Diseases, miRNA AND Cardiovascular Diseases.

In this review, we first describe the definition of miRNAs and their actions at the cellular level. Subsequently, we discuss the role of miRNAs as circulating biomarkers, and their role in cardiac remodeling and atherosclerosis.

### MiRNA biology

For many years, it was believed that non-coding regions of the genome were “junk”, as they did not carry information for protein synthesis. Currently, it is known that most of the eukaryotic transcriptome is composed by noncoding RNAs, which are classified as functional and regulators. Among functional RNAs, there are transfer RNA (tRNA), small nuclear RNA (snRNA) and small nucleolar RNA (snoRNA); major classes of RNAs regulators are miRNAs, small interfering RNAs (siRNAs), piwiRNAs (piRNAs) and long noncoding RNAs (lncRNAs).^5^

Among this wide variety of noncoding RNA classes, much attention has been drawn to miRNAs because of the association between dysregulation of these molecules and development of phenotypic and pathological changes.^6^ MiRNAs are defined as single-stranded, small noncoding RNA molecules containing about 22 nucleotides (nt). They function in post-transcriptional regulation of gene expression in plants and animals by means of cleavage of the target messenger RNA (mRNA) or by suppression of mRNA translation.^7^ The first miRNA, lin-4, was described in 1993 by the group of Rosalind Lee as a miRNA involved in the larval development of *Caenorhabditis* *elegans* (*C. elegans*). Lin-4 negatively regulates the level of LIN-14 protein in the first larval stage, decreasing its expression over time.^1^

**Figure 1:**
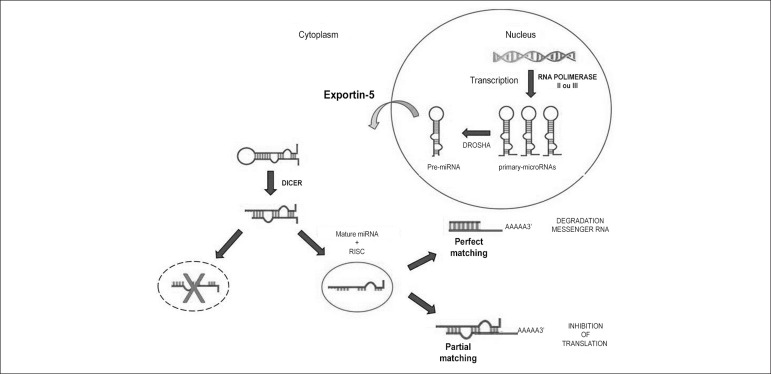
Synthesis of miRNA and its action on messenger RNA (mRNA). Hairpin primary-microRNAs are synthesized in the nucleus, converted into pre-miRNA by Drosha enzyme, and exported from the nucleus into the cytoplasm by the Exportin-5 protein. In the cytoplasm, pre-miRNA is recognized by the enzyme Dicer; RNA-induced silencing complex (RISC) binds to a double-stranded RNA (dsRNA), generating mature miRNA. Mature miRNA interacts with the target mRNA, leading to either its degradation or its translation.

MiRNA biogenesis starts with the synthesis of a long primary transcript, known as pri-miRNA (~110pb) (Figure 1). Pri-miRNAs are transcribed by RNA polymerase II or III;^8^ they contain a hairpin structure, essential for its recognition by miRNA processing enzymes. Pri-miRNA is processed into pre-miRNA (~70pb) by the nuclear RNaseIII enzyme Drosha^9^ which recognizes and cleaves the ends of the hairpin-shaped small RNA structures.^10^

Following the nuclear processing, each pre-RNA is exported into the cytoplasm by the Exportin-5 protein.^11^ Pre-miRNA is recognized by the enzyme Dicer, which cleaves the loop region into a double-stranded RNA (dsRNA:~22pb). This process recruits proteins of the Argonaute protein family to form the RNA-induced silencing complex (RISC).^12^ RISC binds to one of the strands of the dsRNA and generates mature miRNA (canonical miR or miR-5p) which is involved in the regulation of a target mRNA.^13^ The other strand (miR* or miR-3p) is either degraded or involved in the generation of another RISC, acting in the regulation of another target mRNA.^14^

The perfect matching between miRNA and the three prime untranslated region (3'-UTR) of the target mRNA leads to the cleavage of the mRNA and its transfer to mRNA processing bodies (p-bodies) and subsequent degradation.^15^ On the other hand, a partial matching between miRNA and 3’-UTR inhibits translation, which is the main mechanism of action of the miRNAs in mammals.^16^ Thus, by translation inhibition, miRNA has a direct effect on translation factors and on poly(A) tail functioning.^17^ Although the primary location of miRNAs is cell cytoplasm,^8^ some studies have confirmed the entry of these molecules into the circulatory system, possibly resulting from cell lysis.^18^

Therefore, MiRNAs are also found in the circulatory system and several studies have shown its high stability in the extracellular environment. MiRNA degradation in the extracellular milieu could be prevented by its binding to proteins (e.g. lipoproteins) or its encapsulation into microvesicles and exosomes. Thereby, miRNAs can be reliably detected in plasma samples, and suggested as potential biomarkers of CVDs.^19^ Since miRNAs are small sequences and do not require perfect matching, a unique miRNA can have tens of target mRNA, and a unique mRNA can be the target of multiple miRNAs, producing a broad regulatory power of genetic expression.^20^

### MiRNAs as circulating biomarkers

**Figure 2:**
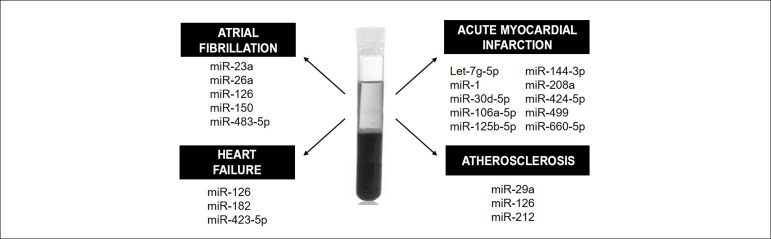
Circulating biomarkers. Some of the serum and plasma miRNAs that could be used as diagnostic or prognostic biomarkers in cardiovascular diseases are here illustrated.

Circulating levels of miRNAs have been shown to be altered in some CVDs. This fact has aroused interest in using it as a diagnostic and prognostic tool, since circulating miRNAs have high stability and are easily detected. MiRNAs could be used, for example, as biomarkers of heart failure (HF), atrial fibrillation, acute myocardial infarction (AMI) and atherosclerosis, by its detection in blood plasma (Figure 2).

It has been well established that the presence of miR-1 in the blood may be helpful in the detection of AMI. However, its long-term use as a biomarker has not been recommended, since it remains circulating in the blood only for a short period. The short half-life of MiR-1 is probably explained by its direct release from cardiac necrotic tissue into the circulation, not encapsulated in exosomes.^21^ In case of heart injury, miRNAs would be released into the blood through exosomes or by cell rupture, associated or not with other molecules. These molecules could protect miRNAs from degradation, prolonging its time in circulation. Besides, the increase in blood flow, changes in pH and release of cytokines can also affect the half-life of circulating miRNAs. For example, it was shown that exogenously added mature miR-1 is rapidly degraded both *in vitro* and *in vivo*.^22^ One limitation of the use of circulating miRNA is the lack of normalization of its quantification as compared with tissue miRNA.^23^ Despite these limitations, miR-1 is highly sensitive in early identifying AMI.

Several studies have suggested a higher diagnostic accuracy of miR-499 compared with troponin T for AMI.^24^^,^^25^ MiR-499 has the advantage of being detectable in the blood within the next four hours after the AMI, whereas troponin can be detected only later.^26^ Therefore, miR-499 could enhance the accuracy of troponin T in the early diagnosis of AMI. The HUNT study investigated 179 miRNAs in 212 healthy subjects aiming to predict AMI in these individuals. Several circulating miRNAs were significantly different between individuals who suffered from fatal AMI and those who remained healthy during the follow-up period. Logistic regression analysis revealed that five miRNAs (miR-106a-5p, miR-424-5p, let-7g-5p, miR-144-3p and miR-660-5p) composed the best model for predicting AMI, providing 77% correct classification for both genders.^27^ Jia et al.^28^ showed that miR-30d-5p and -125b-5p also have diagnostic value for AMI, in a study on acute coronary syndrome patients.^28^ In an animal model of AMI, serum miR-208a was increased at 4 hours and 24 hours after AMI.^25^

Several studies have been conducted aiming at evaluating prognostic and/or diagnostic value of miRNAs in heart failure (HF), as well as in HF treatment. There is evidence that miRNAs have an important role in both the initiation and progression of HF. Although brain natriuretic peptide (BNP) and N-terminal pro-B-type natriuretic peptide (NT-proBNP) are considered the gold standard for HF diagnosis, miRNAs have been exhaustively studied as potential biomarkers. For example, a recent systematic review with meta-analysis showed that miR-423-5p, as associated with atrial natriuretic peptide (ANP) would have a potential diagnostic value for HF detection.^29^ In chronic HF, Cakmak et al.^30^ showed that miR-182 has a higher prognostic value for cardiovascular mortality, characterized by unexplained sudden death, decompensated HF or hemodynamically significant arrhythmia, as compared with NT-proBNP and high-sensitivity C-reactive protein (CRP) by ROC curve analysis in patients with compensated HF (NYHA II, n = 20) and decompensated HF (NYHA III, n = 22) compared with healthy controls (n = 15).^30^ A more comprehensive overview of miRNAs involved in acute and chronic HF can be found in a recent review by Vegter et al.^31^

Studies on atrial fibrillation (AF) patients have shown that patients with stable chronic HF, AF and ejection fraction < 40% show a significant reduction in plasma miR-150 compared with healthy controls.^32^ Harling et al.^33^ analyzed the plasma obtained at 24 h before myocardial revascularization to evaluate diagnostic accuracy of miRNA in post-operative AF. The authors showed a predictive accuracy of 78%,^33^ indicating that miR483-5 is a potential biomarker of post-operative AF. There is evidence that miR-23a and miR-26a could also predict post-operative AF, since their levels are reduced in the postoperative period of patients undergoing coronary bypass artery grafting surgery.^34^ Reduced circulating levels of miR-126 is a potential biomarker of the development, progression and severity of AF and HF, according to a study conducted with patients with AF, HF or both.^35^ Also, Gorem et al.^36^ showed reduced expression of miR-150 in both platelets and serum of patients with chronic systolic HF associated with AF, compared with individuals without AF.^36^

A study^37^ reported the synthesis of endothelial cell-derived apoptotic bodies containing high levels of miR-126 in atherosclerotic vascular disease. These molecules triggered the production of the chemokine CXCL12 in recipient vascular cells.^37^ Circulating miR-212 has been suggested by Jeong et al.^38^ as a biomarker of atherosclerosis, as it improves the prediction of atherosclerosis when combined with hemoglobin A1c, HDL and lipoprotein(a). Elevated plasma miR-29a levels were associated with increased carotid intima-media thickness in atherosclerosis patients.^39^

### Cardiac remodeling

**Figure 3:**
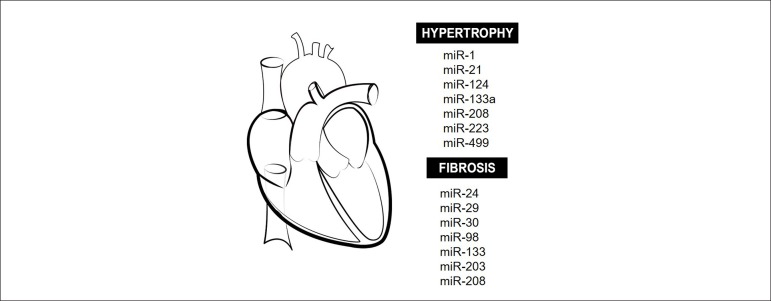
Cardiac remodeling and miRNAs. Main miRNAs that modulate cardiac hypertrophy and tissue fibrosis during adverse cardiac remodeling in many diseases are here illustrated.

Ventricular remodeling is one of the mechanisms involved in the progression of HF and involves cardiomyocyte apoptosis and hypertrophy, interstitial fibrosis caused by collagen deposition, and vascular rarefaction (Figure 3). Despite evidence of an important role of miRNAs on pathologic remodeling, many miRNAs involved in this process remain to be identified. Also, there have been conflicting results on the association of miRNAs with diseases.

MiR-1, -133a, -208a/b and -499 are believed to be specific for cardiac tissue, as they are more abundant in this tissue than in others. These miRNAs are involved in mesodermal precursor differentiation, in transdifferentiation/reprogramming of adult fibroblasts/myofibroblasts into mature cardiomyocytes, and preservation of normal function and survival of cardiomyocytes.^40^ In pathologic conditions, dysregulation of cardiac miRNAs may lead to HF progression, combined with arrhythmia, ischemia, ventricular dilatation, fibrosis and tissue necrosis.

### Cardiomyocytes

MiR-1 is one of the most abundant miRNAs, responsible for the control of different aspects of differentiation and proliferation of cardiomyocytes. In animals, miR-1 would be involved in the proliferation and differentiation of cardiac cells during cardiogenesis.^41^ However, increased expression of miR-1 can cause arrythmia, as it controls cardiac conductance and automaticity by modulating the expression of proteins involved in intracellular calcium regulation.^42^ MiR-21 is predominantly expressed in cardiac fibroblasts. Its increased expression was shown to indirectly promote cardiac hypertrophy by stimulating Mitogen Activated Protein (MAP) kinases in an animal model of HF,^43^ although there is evidence that increased expression of miR-1 has an anti-hypertrophic role in isolated cardiomyocytes.^44^

Silencing of miR-208 in an animal model of AMI attenuated apoptosis, hypertrophy and fibrosis, promoting improvement in cardiac function.^45^ There is evidence that miR-133 protects cardiomyocytes from hypertrophy in neonatal rats. Mechanisms involved in this process include modulation of intracellular calcium concentrations and reduction of mRNA expression into ANP and myosin heavy chain beta MHC-β.^46^^,^^47^ MiR-223 could suppress hypertrophy by decreasing calcium intracellular concentrations, cardiomyocyte contractility, and phosphorylation of cardiac troponin I (cTNI).^4^

MiR-124 would be involved in cardiac hypertrophy, since its expression is increased in a model of angiotensin II-induced hypertrophy in primary cultured rat neonatal cardiomyocytes, and inhibition of its expression would suppress angiotensin II-induced hypertrophy.^49^ In mice, induction of miR-499 expression in the heart caused cellular hypertrophy and cardiac dysfunction due to altered expression of contractile proteins - MYH7B and skeletal muscle actin alpha 1 (ACTA1).^50^

### Fibrosis

Connective tissue growth factor (CTGF) is considered a key molecule in the fibrotic process, as it induces the synthesis of extracellular matrix (ECM). Duisters et al.^51^ demonstrated that miR-133 and miR-30 regulate CTGF expression.^51^ CTGF expression is inversely proportional to the expression of these miRNAs in models of cardiac diseases in rodents (genetic model with hyperactivation of the renin-angiotensin-aldosterone system, cardiac hypertrophy and HF) and in disease-related left ventricular remodeling in biopsy samples of patients with aortic stenosis undergoing valve replacement surgery. Besides, increased expression of miR-133 and miR-30 reduces CTGF expression, resulting in decreased collagen deposition.^51^ On the other hand, miR-203 may play a pro-fibrogenic role, since induction of its expression in cultured mouse cardiomyocytes increases the synthesis of CTGF, transforming growth factor beta 1 (TGF-β1) and fibronectin.^52^

Mir-29 also seems to play an important role in ECM remodeling in patients with HF. Mir-29 is preferentially expressed in fibroblasts, in areas surrounding infarcted areas. It would be involved in apoptosis, specially at final stages of HF, reducing collagen expression.^53^ In an animal model of infarction, miR-24 expression is reduced, which is correlated with EMC remodeling. MiR-24 expression induced by synthetic precursors inhibits fibrosis, and differentiation and migration of cardiac fibroblasts.^21^ MiR-98 seems to have a similar mechanism, since induction of its expression in human cardiac fibroblasts inhibits TGF-β1-induced fibrosis.^54^

### Tissue expression profile based on diseases

Ikeda et al.^55^ performed a broad analysis of miRNA expression in 67 samples of left ventricular myocardium in ischemic cardiomyopathy, dilated cardiomyopathy and aortic stenosis patients. They found distinct microRNA expression profiles according to different diseases; expression of 13 miRNAs was specific to aortic stenosis, and 8 miRNAs specific to both cardiomyopathies, with no overlapping between both groups.^55^ In dilated cardiomyopathy and aortic stenosis, expression of miR-1, -19a and -19b was reduced and miR-214 expression was increased; this was related to cardiac hypertrophy, with no changes in miRNA-133 and -208 expression.^55^ Nevertheless, Care et al.^56^ showed reduced expression of miRNA-133 in hypertrophic cardiomyopathy and atrial dilation, whereas Yang et al.^57^ reported increased expression of miR-1 in ischemic cardiomyopathy.

In the study by Lai et al.,^53^ the association of several miRNAs with HF was investigated in biopsy specimens taken from left ventricular apex during cardiac surgery. Increased expression of miR-1, -21, -23, -29, -130, -195 and -199 was found in myocardium of these patients, whereas miR-30, -133 and -208 expression was unchanged. This was associated with higher mRNA expression for caspase-3, type I and type III collagen and TGF.^53^

### Atherosclerosis

**Figure 4:**
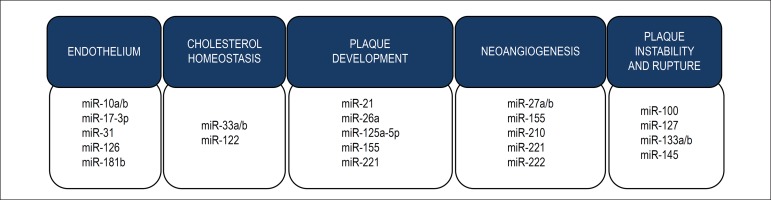
Atherosclerosis and miRNAs. Dysregulation of the expression of several miRNAs has been found in different stages of atherosclerosis formation. Some of the miRNAs involved in endothelial dysfunction and inflammation, cholesterol homeostasis, plaque development, neoangiogenesis and plaque instability and rupture are here illustrated.

Atherosclerosis is a chronic inflammatory disease of the artery walls in response to endothelial injury, especially in medium and large sized elastic vessels, muscular arteries and regions with disturbed laminar blood flow. It is considered the main cause of coronary artery disease, carotid artery disease, stroke and peripheral vascular disease.^58^ Several evidences have shown the involvement of miRNAs in the development of atherosclerosis, in both human and animal models. MiRNAs can be categorized into miRNAs involved in endothelial dysfunction, cholesterol homeostasis, development of atherosclerotic plaque, neoangionesis and plaque instability and rupture, as described in Figure 4.

### Endothelium

In pigs, endothelial cells from regions susceptible to atherosclerosis (aortic arch and abdominal aortic-renal artery bifurcation) showed reduced expression of miR-10a and -10b. MiR-10a inhibits some pro-inflammatory genes in endothelial cells, including vascular cell adhesion molecule-1 (VCAM-1) and E-selectin, as well as the NF-kB pathway.^59^ In rats, miR-181b regulates endothelial cell activation and vascular inflammatory response to NF-kB in the presence of pro-inflammatory stimuli.^60^ In human umbilical vein endothelial cells (HUVEC), miR-126, miR-31 and miR-1703p also regulate vascular inflammation by controlling the expression of cell adhesion molecules - VCAM-1, intercellular adhesion molecule-1 (ICAM-1) and E-selectin.^61^

### Cholesterol homeostasis

MiR-33a and miR-33b regulate SREBP2 and SREPB1 genes, responsible for cholesterol regulation and fatty acid metabolism, in human and mice cells.^62^ Inhibition of miR-33a inhibited atherosclerosis in mice.^63^ Inhibition of miR-122 expression, which accounts for 80% of miRNAs expressed in the liver, significantly decreased cholesterol serum levels in mice and non-human primates.^64^

### Plaque development

MiR-155 is an important regulator of the immune system and seems to be involved in acute inflammatory response. MiR-155 modulates the development of atherosclerotic plaque, lipid uptake and the inflammatory response of monocytes and macrophages that leads to foam cell formation. Among its mechanism of action, this miRNA acts as a regulator of the negative feedback in oxidized LDL-induced inflammatory response in macrophages and inhibits the release of inflammatory cytokines from macrophages, such as interleukin 6 (IL-6) and IL-8 and tumoral necrosis factor alpha (TNF-α).^65^

In peripheral blood monocytes in humans, miR-125a-5p showed an important role in mediating lipid absorption and reducing the secretion of some inflammatory cytokines (IL-2, IL-6, TNF-α and TGF-β) in macrophages.^66^ Oxidized LDL increased the levels of miR-125a-5p, which regulates oxysterol-binding protein-related protein (ORP)-9, hence decreasing the expression of scavenger receptors (CD68) and LOX-1.^66^ Similarly, miR-155 reduced oxidized LDL uptake, decreasing the expression of CD36 and LOX-1.^65^

Progression of fatty streaks to fibrous cap of an atheroma is mainly caused by proliferation and migration of vascular smooth muscle cells (VSMCs) to the intima. Proliferation and apoptosis of these cells are regulated by TGF-β, which, in turn, is negatively regulated by miR-26a (i.e., miR-26 inhibition promotes VSMC differentiation) in human serum.^67^ In addition, both miR-21 and miR-221 also modulate the proliferation of VSMC; miR-221 acts in response to platelet derived growth factor (PDGF). Also, miR-221 negatively regulates p2Kip1, which is critical for induction of cell proliferation mediated by PDGF, whereas c-Kit may be associated with inhibition of VSMC-specific contractile gene transcription by reducing the expression of myocardin, a potent VSMC-specific nuclear coactivator.^68^

### Neoangionesis

During the development of atherosclerotic plaque, activated, cholesterol-containing macrophages are responsible for the release of several cytokines, including those involved in neoangiogenesis. MiRNAs involved in this process include miR-221, -222, -155, -27a, -27b and -210. In HUVEC cells, miR-222/221 affect the expression of c-Kit,^69^ and miR-222 is involved in vascular remodeling mediated by inflammation.^70^ MiR-155 seems to regulate the expression of endothelial nitric oxide synthase (eNOS) and endothelium-dependent vascular relaxation.^71^ In a three-dimensional spheroid model, increased expression of miR-27a/b stimulates endothelial cell sprouting, indicating its pro-angiogenic effect, since they target semaphorin 6A, an angiogenesis inhibitor.^72^ Finally, miR-210 expression in HUVEC progressively increases in hypoxia and its increased expression in normoxia leads to formation of capillary-like structures by VEGF on Matrigel.^73^

### Plaque instability and rupture

Instability and rupture of the fibrous capsule of an atherosclerotic plaque depend on the balance between synthesis and degradation of the ECM by fibroblasts. Plaque rupture is the main mechanism involved in the development of stroke and AMI, and sudden death. Matrix metalloproteinases (MMPs) act in collagen degradation, especially MMP-1, MMP-2, MMP-3 and MMP-9 released by activated macrophages.^74^

MMP-9 is regulated by miR-133a/b, which can also modulate VSMC apoptosis and proliferation in animal models.^75^ Cipollone et al.^76^ investigated miRNA expression and its correlation with plaque instability in internal carotid artery in humans. Two independent cohorts of atherosclerotic plaques of patients who underwent carotid endarterectomy for extracranial high-grade (>70%) internal carotid artery were collected in two Italian hospitals (n = 15 and n = 38). The plaques were subdivided into 2 groups (symptomatic and asymptomatic plaques) according to the presence or absence of stroke. The authors observed that, among the 41 miRNAs examined, there was increased expression of 5 miRNAs (miNA-100, miRNA-127, miRNA-145, miRNA-133a, and miRNA-133b) in symptomatic compared with asymptomatic plaques.^76^ It is worth mentioning that differences in the expression of miRNAs between stable and unstable plaques were not related to differences in conventional risk factors or concomitant therapies, since these variables were well balanced between the two groups. Incubation of HUVECs with miR-133 downregulated the expression of plasminogen activator inhibitor-1 (PAI-1).^76^

## Conclusion

Despite all difficulties and challenges, it is crucial to identify dysregulated miRNAs, as it allows a better understanding of cellular and molecular mechanisms involved in CVDs. Studies on tissue and circulating miRNAs could help in the development of more accurate diagnostic and prognostic circulating markers, as well as new therapeutic strategies for different stages of CVDs.

Despite advances in this field, there are still some limitations, for example, in using circulating miRNAs as biomarkers. Molecular processes that control the packing and release of extracellular miRNAs have not been fully elucidated, including mechanisms mediated or not by vesicles. Besides, detection of circulating miRNAs requires high technical skills, which could limit their use in routine laboratory use. Another limiting factor is the omnipresence of miRNAs in the circulation, requiring further investigations to identify its tissue origin.
